# Conspectus Medicinæ Theoreticæ. Ad usum Academicum

**Published:** 1784

**Authors:** 


V. *Conſpectus Medicinæ Theoreticæ. Ad uſum Academicum. Auctore* Jacobo Gregory, M. D. *Med. Theoretic. in Acad. Edin. Prof. Col. Reg. Med. Edin. Soc. Societ. Reg. Med. Edin. Sod. Honor. et Societ. Philoſ. Edin. Sod. Ejuſdemque a ſecretis. Editio altera auctior et Emendatior.* 8vo. Creech, *Edinburgh*, 2 vols.

THIS work is intended as a text-book for the author's lectures. In the former edition, Dr. Gregory confined himſelf to Phyſiology and Pathology, which he combined in ſuch a manner, that after deſcribing the nature and uſes of any particular organ or part of the body, he proceeded to treat of its diſeaſes. The ſame judicious arrangement is obſerved in the preſent present edition; but this part of his work is considerably enlarged and improved, and he has now added a second part, *de Therapeia*, which fills almost the whole of the second volume; so that the work, in its present form, whether we consider the value of the materials of which it is composed, or the clear and elegant style in which it is written, cannot be too warmly recommended to the notice of the medical student.

In a preface, which extends through the first 70 pages, the learned author treats of medical theory in general, and gives a concise but interesting account of the principal sects that have prevailed in Physic. In this part of his work he introduces a lively, but, we fear, too true a picture of the uncouth and barbarous style that distinguishes too many of the pseudo-latin writers, as he calls them, of the present age; and at the same time he takes occasion to point out the disadvantages resulting to medical science from the present too general disuse of the Latin language, the cultivation of which he recommends with great earnestness to his readers. His observations on this head are certainly well founded; yet we are persuaded that the ingenious author, by permitting his work work to appear in an English dreſs, will render it much more extenſively uſeful, than it can otherwiſe be, in this country.

